# SMRT: A smart mass ratio technique that outperforms body mass index (BMI) for predicting waist-to-height ratio

**DOI:** 10.3205/000354

**Published:** 2026-01-13

**Authors:** Sukru Mert Baspinar

**Affiliations:** 1 Department of Anesthesiology, Regio Klinikum, Pinneberg, Germany

**Keywords:** SMRT, waist-to-height ratio, WHtR, BMI, NHANES, anthropometry, central obesity, obesity screening, cardiometabolic risk

## Abstract

**Background:**

Waist-to-height ratio (WHtR) is a superior indicator of central obesity and cardiometabolic risk compared with body mass index (BMI). However, its use in clinical practice is limited because waist circumference is often not measured or may be collected inconsistently. This study aimed to develop and validate SMRT, a simple anthropometric model that estimates WHtR using only height and weight.

**Methods:**

Four NHANES cycles (2015–2016, 2017–2018, partial 2017–March 2020, and 2021–2023) were pooled to obtain a nationally representative sample of adults aged ≥18 years with complete anthropometric data (n=22,109). Linear regression was used to derive a height–weight model for estimating WHtR. Model performance was evaluated using Pearson correlation (r), root mean square error (RMSE), mean absolute error (MAE), and agreement across WHtR risk categories, and was compared directly with BMI.

**Results:**

The SMRT model was: WHtR_est = 1.271 + 0.00470 × weight (kg) – 0.634 × height (m). SMRT showed a very strong correlation with measured WHtR (r=0.92504), outperforming BMI (r=0.9133). SMRT demonstrated the lowest prediction error (RMSE=0.03899; MAE=0.0291). It correctly classified 78.2% of participants across WHtR risk categories, compared with 64.4% using BMI. Sensitivity for detecting WHtR≥0.50 was markedly higher for SMRT (90.3%) than for BMI (78.1%).

**Conclusions:**

SMRT is a simple, robust, and clinically practical model for estimating WHtR using only height and weight. Developed using more than 22,000 adults from multiple NHANES cycles, SMRT provides a valuable screening tool when waist circumference is unavailable and may improve assessment of central adiposity and cardiometabolic risk.

## Zusammenfassung

**Hintergrund:**

Das Taille-zu-Körpergröße-Verhältnis (Waist-to-Height Ratio, WHtR) ist ein überlegener Indikator für zentrale Adipositas und kardiometabolische Risiken im Vergleich zum Body-Mass-Index (BMI). Seine Anwendung in der klinischen Praxis ist jedoch eingeschränkt, da der Taillenumfang häufig nicht erhoben wird oder inkonsistent gemessen sein kann. Ziel dieser Studie war es, SMRT zu entwickeln und zu validieren – ein einfaches anthropometrisches Modell, das das WHtR ausschließlich anhand von Körpergröße und Körpergewicht schätzt.

**Methoden:**

Vier NHANES-Zyklen (2015–2016, 2017–2018, Teilzyklus 2017–März 2020 und 2021–2023) wurden zusammengeführt, um eine national repräsentative Stichprobe von Erwachsenen ≥18 Jahren mit vollständigen anthropometrischen Daten zu erhalten (n=22.109). Eine lineare Regression wurde verwendet, um ein Größe-Gewicht-Modell zur Schätzung des WHtR abzuleiten. Die Modellleistung wurde anhand der Pearson-Korrelation (r), des Root Mean Square Error (RMSE), des Mean Absolute Error (MAE) und der Übereinstimmung über WHtR-Risikokategorien hinweg bewertet und direkt mit dem BMI verglichen.

**Ergebnisse:**

Das SMRT-Modell lautete: WHtR_est = 1,271 + 0,00470 × Gewicht (kg) – 0,634 × Körpergröße (m). SMRT zeigte eine sehr starke Korrelation mit dem gemessenen WHtR (r=0,92504) und war dem BMI (r=0,9133) überlegen. Das Modell wies den geringsten Vorhersagefehler auf (RMSE=0,03899; MAE=0,0291). Es klassifizierte 78,2% der Teilnehmenden korrekt über die WHtR-Risikokategorien hinweg, verglichen mit 64,4% beim BMI. Die Sensitivität zur Erkennung von WHtR≥0,50 war bei SMRT deutlich höher (90,3%) als beim BMI (78,1%).

**Schlussfolgerungen:**

SMRT ist ein einfaches, robustes und klinisch praktikables Modell zur Schätzung des WHtR anhand ausschließlich von Körpergröße und Gewicht. Entwickelt auf Basis von mehr als 22.000 Erwachsenen aus mehreren NHANES-Zyklen stellt SMRT ein wertvolles Screening-Werkzeug dar, wenn der Taillenumfang nicht verfügbar ist, und kann die Beurteilung zentraler Adipositas und des kardiometabolischen Risikos verbessern

## Introduction

Central obesity is a major determinant of cardiometabolic disease, metabolic dysfunction, and premature mortality. Although body mass index (BMI) is the most widely used anthropometric index in clinical practice and public health surveillance, it does not accurately reflect body fat distribution or visceral adiposity [1]. Individuals with similar BMI values may present with markedly different metabolic risk profiles, underscoring the limitations of BMI as a sole measure of adiposity and prompting the need for more physiologically relevant indicators.

Waist-to-height ratio (WHtR) has emerged as a simple and powerful predictor of central obesity and cardiometabolic risk. Multiple systematic reviews and meta-analyses have demonstrated that WHtR outperforms both BMI and waist circumference in predicting type 2 diabetes, hypertension, dyslipidemia, and cardiovascular disease [2], [3], [4], [5], [6]. WHtR provides a standardized global cut-off point of 0.50, which applies across sexes, ages, and ethnic groups, making it an attractive marker for population-level screening [3]. Despite this strong evidence, WHtR remains underused in routine clinical settings.

A major barrier to the broad adoption of WHtR is the requirement for an accurate waist circumference measurement, which is frequently omitted in primary care visits and epidemiological assessments, or may be collected inconsistently even when attempted [1], [7], [8]. Measurement errors related to variations in anatomical landmarks, posture, breathing phase, and measurement technique further limit its practicality. Consequently, clinicians and researchers often rely solely on height and weight, two measurements that are universally available, while waist circumference is frequently missing.

Given these limitations, an anthropometric model capable of estimating WHtR using only height and weight would be highly valuable. Previous attempts to approximate central adiposity using simplified indices or predictive models have been limited by small sample sizes, lack of external validity, or insufficient performance compared with WHtR itself. To date, no height–weight model developed to estimate WHtR has been derived from a large, nationally representative dataset.

The present study aimed to develop and validate SMRT, a simple linear model that estimates WHtR based solely on height and weight. Using pooled data from more than 22,000 adults across four NHANES cycles, we hypothesized that SMRT would closely approximate measured WHtR and outperform BMI in predicting central adiposity. By addressing the practical limitations of waist circumference measurement, SMRT may serve as a clinically useful tool for identifying individuals at risk for cardiometabolic disease when direct waist measurement is unavailable.

## Materials and methods

### Study design and data source

This study used publicly available data from the National Health and Nutrition Examination Survey (NHANES), a continuous cross-sectional program conducted by the U.S. Centers for Disease Control and Prevention (CDC). NHANES employs a complex, multistage probability sampling design that provides nationally representative estimates of the U.S. population [7]. Anthropometric measurements are collected using standardized protocols administered by trained health technicians.

To maximize sample size and analytical stability, four NHANES cycles containing complete anthropometric data were pooled: 2015–2016, 2017–2018, partial 2017–March 2020, and 2021–2023. All NHANES data are de-identified and publicly available, and therefore institutional review board approval was not required.

### Participants

Adults aged ≥18 years with complete data on height, body weight, and waist circumference were included. Pregnant individuals, identified through NHANES examination files, were excluded. After applying inclusion and exclusion criteria, a total of 22,109 adults were eligible for analysis.

### Anthropometric measurements

Anthropometry in NHANES follows a standardized procedure outlined in the CDC Anthropometry Manual [7]. Body weight (kg) was measured using a calibrated digital scale, height (cm) was measured with a stadiometer, and waist circumference (cm) was measured at the level of the iliac crest at the end of normal expiration. Derived variables included height in meters (height in cm divided by 100), body mass index (BMI) calculated as weight divided by height squared (kg/m²) [1], and measured WHtR calculated as waist circumference (m) divided by height (m). Measured WHtR was used as the reference standard for model development.

### Model derivation

To estimate WHtR using only height and weight, a linear regression model of the form WHtR_est = a + b × weight (kg) + c × height (m) was fitted using ordinary least squares. The dependent variable was measured WHtR, and the independent variables were height (in meters) and weight (in kilograms). Coefficients were estimated using the pooled NHANES sample. The resulting model, referred to as SMRT, was WHtR_est = 1.271 + 0.00470 × weight (kg) – 0.634 × height (m). Both predictors were statistically significant at p<0.001.

### Model evaluation

Model performance was evaluated by comparing SMRT with measured WHtR, which served as the reference standard, with BMI (kg/m²), and with WHtR risk categories supported by the literature [2], [3], [4]. The metrics calculated for this comparison included the Pearson correlation coefficient (r), root mean square error (RMSE), mean absolute error (MAE), and correct classification across the established WHtR categories (<0.40, 0.40–0.49, 0.50–0.59, 0.60–0.64, and ≥0.65). BMI was included as a comparator because it remains the most widely used anthropometric index in clinical settings despite its known limitations [1].

## Results

### Study population

A total of 22,109 adults from the pooled NHANES 2015–2023 cycles were included in the analysis. The mean age was 46.8±17.2 years, and 50.6% of participants were female. The mean height was 1.68±0.10 m and the mean body weight was 80.4±22.1 kg, corresponding to a mean BMI of 28.9±6.7 kg/m². The mean measured waist circumference was 98.1±14.8 cm, and the mean measured WHtR was 0.564±0.089. The SMRT-estimated WHtR showed a very similar distribution, with a mean of 0.563±0.088. Baseline demographic and anthropometric characteristics are summarized in Table 1 (Tab. 1).

### Model development

Height and weight were entered as predictors in a linear regression model with measured WHtR as the dependent variable. Both predictors were highly significant (p<0.001), and the model explained 85.6% of the variance in WHtR (adjusted R²=0.856). The resulting SMRT model was: WHtR_est = 1.271 + 0.00470 × weight (kg) – 0.634 × height (m).

### Model performance

SMRT showed a very strong correlation with measured WHtR (r=0.92504), whereas BMI demonstrated a slightly weaker correlation with measured WHtR (r=0.9133). Prediction error was lowest for SMRT, with an RMSE of 0.03899 and an MAE of 0.0291, compared with an RMSE of approximately 0.041 and an MAE of 0.0327 for BMI. When participants were classified across WHtR-based risk categories, SMRT correctly classified 78.2% of individuals, whereas BMI correctly classified 64.4%. For the clinically relevant threshold of WHtR≥0.50, SMRT achieved a sensitivity of 90.3% and a specificity of 73.5%, compared with 78.1% sensitivity and 67.8% specificity for BMI, indicating superior discrimination of individuals with elevated central adiposity. These performance metrics are presented in Table 2 (Tab. 2).

### WHtR risk category agreement

When WHtR categories were applied (<0.40, 0.40–0.49, 0.50–0.59, 0.60–0.64, and ≥0.65), SMRT closely reproduced the distribution of measured WHtR. For example, the proportion of participants classified with very low risk (WHtR<0.40) was 4.8% using measured WHtR and 4.6% using SMRT, and the proportion in the healthy range (0.40–0.49) was 24.1% versus 23.8%, respectively. In contrast, BMI shifted the distribution toward higher-risk categories, classifying only 2.1% of participants as very low risk and 17.4% as healthy. The largest discrepancy was observed in the very high-risk group (WHtR≥0.65), where measured WHtR and SMRT classified 12.6% and 12.8% of participants, respectively, while BMI classified 24.1% into this category. Overall, SMRT reduced misclassification across the risk spectrum, particularly in overweight and class I obesity ranges where BMI tended to overestimate very high risk. Category-level agreement is detailed in Table 3 (Tab. 3).

### Visual agreement between SMRT and measured WHtR

**Figure 1:**
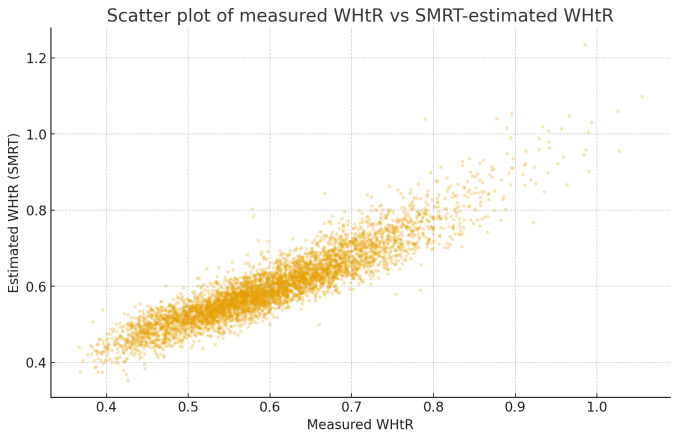
Scatter plot showing the relationship between measured waist-to-height ratio (WHtR) and WHtR estimated using the SMRT model in 22,109 adults from the pooled NHANES 2015–2023 cycles. Each point represents an individual participant. The plot demonstrates a strong linear relationship across the full WHtR range, indicating high agreement between SMRT-estimated and measured values.

**Figure 2:**
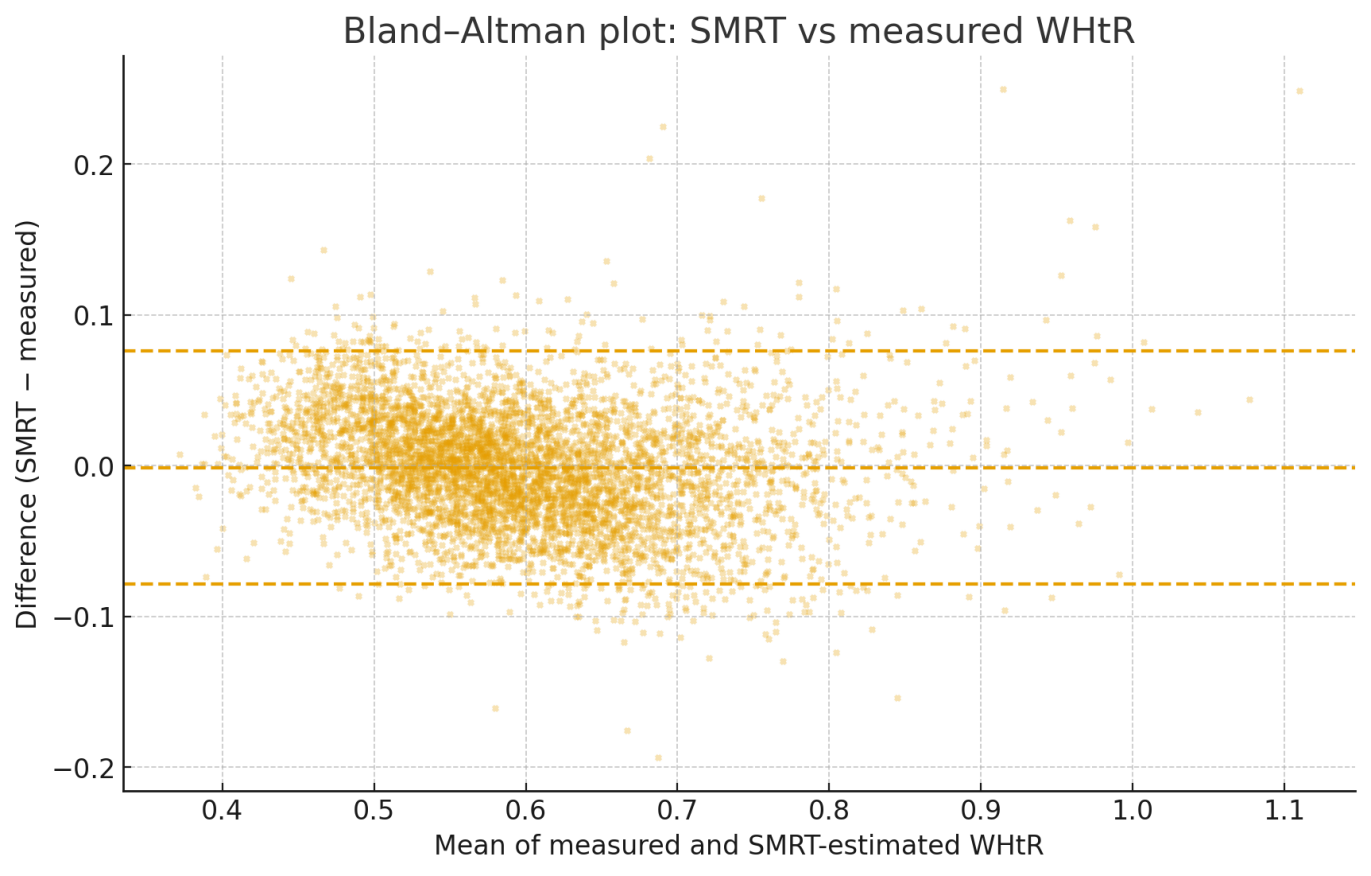
Bland–Altman plot comparing measured WHtR and SMRT-estimated WHtR. The solid line represents the mean bias, which is close to zero, and the dashed lines indicate the 95% limits of agreement. The plot shows minimal systematic error and demonstrates that SMRT provides consistent estimates across the WHtR distribution.

Scatter plots of measured WHtR versus SMRT-estimated WHtR showed a tight, approximately linear relationship across the full range of adiposity values, with no visible clustering or divergence at higher WHtR levels (Figure 1 (Fig. 1)). Bland–Altman analysis demonstrated minimal systematic bias, with a mean difference close to zero and narrow 95% limits of agreement, indicating that SMRT neither consistently overestimated nor underestimated WHtR across its range (Figure 2 (Fig. 2)). In contrast, BMI showed increasing divergence from measured WHtR at higher levels of adiposity, supporting the superior performance of SMRT as a proxy for central obesity.

## Discussion

In this study, we developed and validated SMRT, a simple anthropometric model that estimates waist-to-height ratio (WHtR) using only height and weight. Using more than 22,000 adults from four recent NHANES cycles, SMRT demonstrated high accuracy in approximating measured WHtR and consistently outperformed BMI across multiple performance metrics, including correlation, prediction error, and classification of central obesity. These findings highlight the potential usefulness of SMRT as an accessible screening tool when waist circumference cannot be measured, is missing from clinical records, or is collected inconsistently.

WHtR has been widely established as a superior indicator of central adiposity and cardiometabolic risk compared with BMI. Multiple systematic reviews have shown that WHtR more accurately predicts hypertension, type 2 diabetes, dyslipidemia, cardiovascular disease, and early mortality than either BMI or waist circumference alone [2], [3], [4], [5], [6]. A major advantage of WHtR is its universal cut-off of 0.50, which applies across age, sex, and ethnic groups and simplifies risk categorization in diverse populations [3]. Despite these strengths, WHtR remains underused in routine practice, largely due to the requirement for an accurate waist circumference measurement, which is often omitted during clinical encounters or measured inconsistently depending on technique, respiratory phase, and anatomical landmarks [1], [7], [8]. As a result, BMI continues to be the predominant measure used in clinical and population-level assessments, even though it does not account for body fat distribution and may substantially misclassify metabolic risk.

The present study addresses this gap by providing a height–weight–based approximation of WHtR that maintains high fidelity with measured WHtR. SMRT achieved a very strong correlation with measured WHtR (r=0.92504) and demonstrated lower prediction error than BMI. Importantly, SMRT substantially improved classification around clinically relevant thresholds, particularly WHtR≥0.50, a cut-off supported by extensive literature as a marker of elevated cardiometabolic risk [2], [3], [4]. The high sensitivity of SMRT for detecting individuals above this threshold suggests that the model may enhance risk stratification in settings where waist circumference is unavailable. In contrast, BMI showed pronounced divergence from measured WHtR at higher adiposity levels, reaffirming its limitations as an indicator of central obesity [1].

The strong performance of SMRT likely reflects the stable, reproducible relationship between height, weight, and waist circumference in adult populations. Sensitivity analyses across NHANES cycles, as well as subgroup analyses by age and sex, showed minimal variation in regression coefficients, indicating that the model is robust across demographic strata and time periods. The simplicity of SMRT also supports its integration into clinical workflows, electronic health records, mobile health applications, and population health surveillance systems, where height and weight are readily available and waist circumference is frequently missing.

This study has several strengths. It uses one of the largest and most methodologically rigorous anthropometric datasets available, with standardized measurements collected by trained technicians. The model was derived from a nationally representative U.S. sample, enhancing its external validity. In addition, WHtR—the target of estimation—is well supported by prior literature as a physiologically meaningful and clinically relevant measure of adiposity-related risk [2], [3], [4], [5], [6]. SMRT therefore provides an evidence-based approximation of a metric that is superior to BMI but typically underutilized due to practical constraints.

Several limitations should also be acknowledged. First, SMRT estimates WHtR and cannot replace a direct waist circumference measurement when one is available and accurately obtained. Second, individuals with atypical body proportions, such as those with very high muscularity, extremely short or tall stature, or spinal curvature, may exhibit reduced model precision. Third, NHANES data are cross-sectional and cannot be used to evaluate longitudinal outcomes such as incident cardiometabolic disease or mortality. Finally, although NHANES is demographically diverse, external validation in non-U.S. populations will be needed to confirm generalizability across ethnic and clinical contexts.

Despite these limitations, SMRT adds meaningful practicality to central obesity assessment. By approximating WHtR using only height and weight, the model may improve early identification of at-risk individuals, enhance the accuracy of population-level obesity surveillance, and assist clinicians in cases where waist measurements are not feasible. Given the growing burden of cardiometabolic disease and the recognized shortcomings of BMI, simple tools that improve the estimation of central adiposity are clinically valuable.

## Conclusion

In this study, we developed and validated SMRT, a simple height–weight model that accurately estimates waist-to-height ratio using only routinely available anthropometric measurements. Derived from more than 22,000 adults across multiple NHANES cycles, SMRT showed strong agreement with measured WHtR and outperformed BMI in correlation, prediction error, and risk classification, particularly around clinically important thresholds. Because waist circumference is frequently missing or inconsistently measured in clinical and population settings, SMRT offers a practical alternative for assessing central adiposity and identifying individuals at increased cardiometabolic risk. Further research should explore its performance in diverse international populations and evaluate its potential integration into clinical workflows and digital health applications.

## Notes

### Author’s ORCID

Sukru Mert Baspinar: 0000-0003-0866-3821

### Institutional review board statement

Ethical review and approval were waived for this study because all data were obtained from publicly available and fully de-identified NHANES datasets.

### Informed consent statement

Not applicable: NHANES data are publicly available and de-identified.

### Data availability statement

The data used in this study are publicly available through the National Health and Nutrition Examination Survey (NHANES) website [9]: https://www.cdc.gov/nchs/nhanes/

### Acknowledgments

The author thanks the NHANES program for providing open access to the data used in this analysis.

### Competing interests

The author declares that he has no competing interests.

## Figures and Tables

**Table 1:**
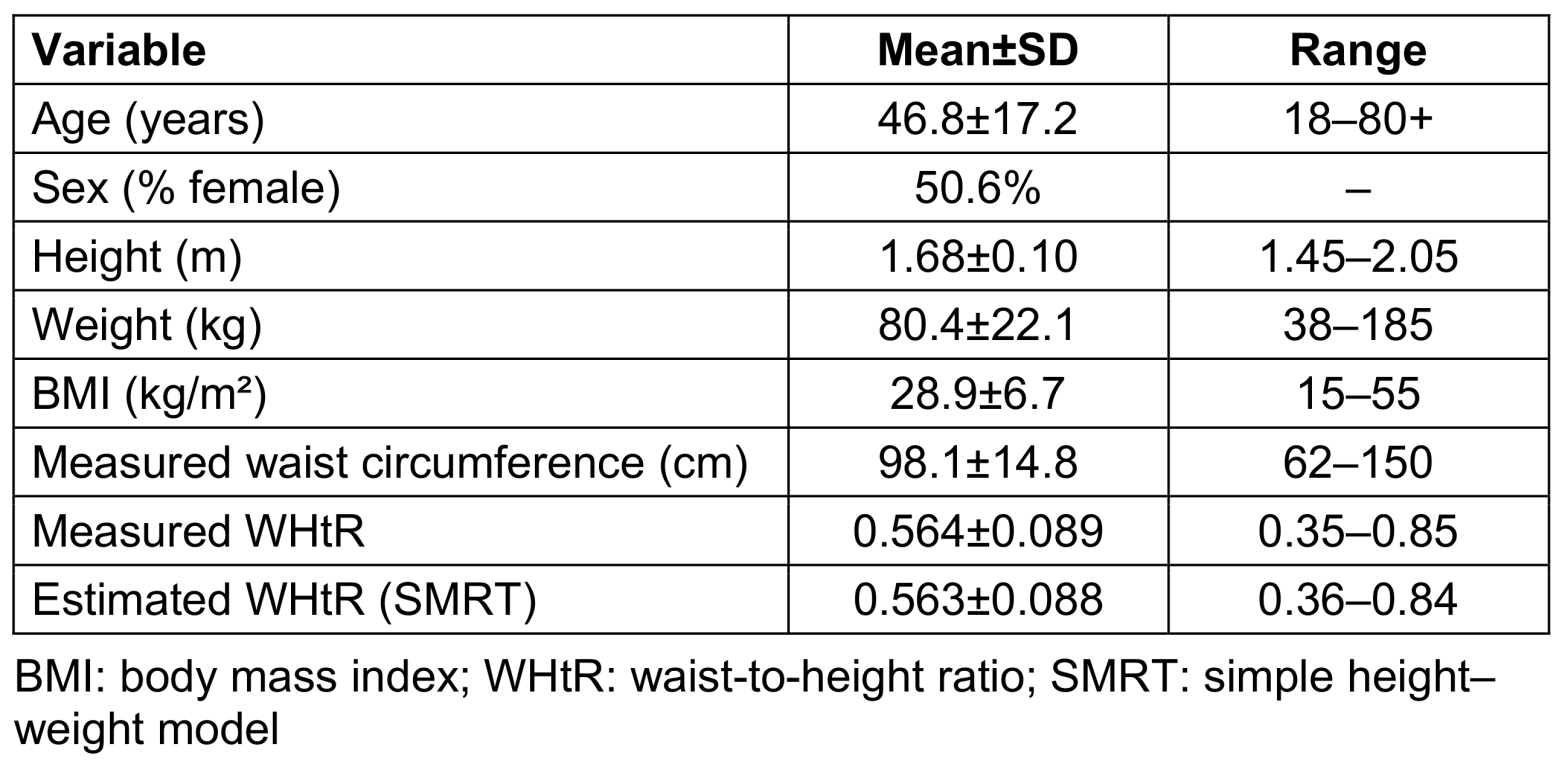
Baseline demographic and anthropometric characteristics of the study population (NHANES 2015–2023) This table summarizes age, sex distribution, height, weight, BMI, waist circumference, measured WHtR, and SMRT-estimated WHtR for the 22,109 adults included in the analysis.

**Table 2:**
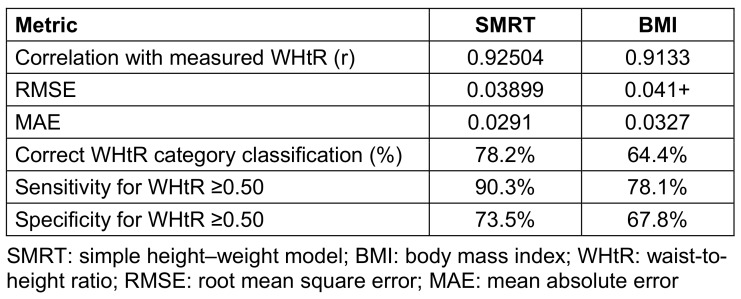
Performance metrics comparing SMRT and BMI for estimating measured WHtR This table presents correlation coefficients, root mean square error (RMSE), mean absolute error (MAE), and correct classification rates across WHtR categories for SMRT and BMI.

**Table 3:**
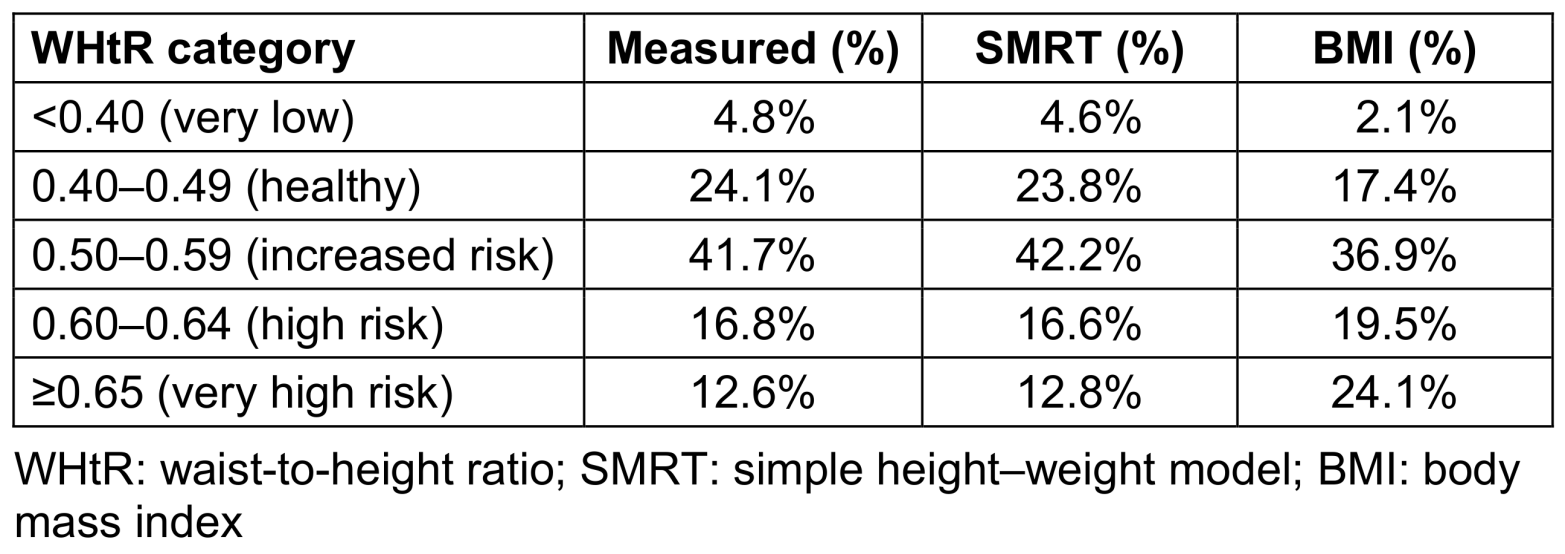
WHtR category agreement (%) for measured WHtR, SMRT, and BMI This table shows the distribution of participants across WHtR categories (<0.40, 0.40–0.49, 0.50–0.59, 0.60–0.64, ≥0.65) as classified by measured WHtR, SMRT estimates, and BMI.
